# Association between the Charlson Comorbidity Index and the risk of 30-day unplanned readmission in patients receiving maintenance dialysis

**DOI:** 10.1186/s12882-019-1538-0

**Published:** 2019-10-07

**Authors:** Yu Lin, Chao Yang, Hong Chu, Jingyi Wu, Ke Lin, Ying Shi, Haibo Wang, Guilan Kong, Luxia Zhang, on behalf of China Kidney Disease Network Working Group

**Affiliations:** 1 Department of Epidemiology and Biostatistics, School of Public Health, Peking University, Beijing, 100191 China; 2 National Institute of Health Data Science, Peking University, Beijing, 100191 China; 3 Center for Data Science in Health and Medicine, Peking University, Beijing, 100191 China; 4 Renal Division, Department of Medicine, Peking University First Hospital; Peking University Institute of Nephrology, Beijing, 100034 China; 5 China Standard Medical Information Research Center, Shenzhen, 518042 Guangdong China; 6 Clinical Trial Unit, First Affiliated Hospital of Sun Yat-Sen University, Guangzhou, 510080 Guangdong China

**Keywords:** 30-Day Readmission, Comorbidity, Charlson Comorbidity Index, Hemodialysis, Peritoneal dialysis

## Abstract

**Background:**

Patients receiving maintenance hemodialysis (HD) and peritoneal dialysis (PD) are frequently hospitalized. Reducing unplanned 30-day hospital readmissions is a key priority for improving the quality of health care. The purpose of this study was to assess the association between the Charlson Comorbidity Index (CCI), which has been used to evaluate multi-comorbidities status, and 30-day readmission in patients on HD and PD therapy.

**Methods:**

The Hospital Quality Monitoring System (HQMS), a national administrative database for hospitalized patients in China was used to extract dialysis patients admitted from January 2013 to December 2015. The outcome was the unplanned readmission following the hospital discharge within 30 days. For patients with multiple hospitalizations, a single hospitalization was randomly selected as the index hospitalization. A cause-specific Cox proportional hazard model was utilized to assess the association of CCI with readmission within 30 days.

**Results:**

Of the 124,721 patients included in the study, 19,893 patients (16.0%) were identified as experiencing unplanned readmissions within 30 days. Compared with patients without comorbidity (CCI = 2, scored for dialysis), the risk of 30-day readmission increased with elevated CCI score. The hazards ratio (HR) for those with CCI 3–4, 5–6 and > 6 was 1.01 (95% confidence interval [CI] 0.98–1.05), 1.09 (95% CI 1.05–1.14), and 1.14 (95% CI 1.09–1.20), respectively.

**Conclusions:**

Our study indicated that CCI was independently associated with the risk of 30-day readmission for patients receiving dialysis including HD and PD, and could be used for risk-stratification.

**Electronic supplementary material:**

The online version of this article (10.1186/s12882-019-1538-0) contains supplementary material, which is available to authorized users.

## Background

End-stage kidney disease (ESKD) is a leading cause of morbidity and mortality worldwide [1]. Compared with patients with chronic diseases but without ESKD [2], patients receiving maintenance hemodialysis (HD) and peritoneal dialysis (PD) tend to have a shorter life expectancy, as well as a higher rate of hospitalization and readmission. In the United States, it was observed that 35.2% of patients on HD and PD were readmitted within 30 days in 2012, which drew the attention of the US Centers for Medicare and Medicaid Services [3]. Starting in 2017, the 30-day readmission has been included in the ESKD Quality Incentive Program as an important move in payment reform [4]. A subsequent 2% reduction of overall payment of dialysis patients was observed after the implementation of the program [5], which indicated that 30-day readmission is an important indicator for the quality of healthcare among HD and PD patients.

Previous studies revealed that multiple comorbidities were associated with the risk of 30-day readmission for maintenance HD patients [6, 7]; though it is difficult to quantitatively or semi- quantitatively use those results in clinical practice for risk-stratification. The Charlson Comorbidity Index (CCI) is the most frequently used tool to measure co-existing diseases [8], and it has been validated for predicting the risk of mortality, disability, hospitalization and length of hospital stay in various clinical settings [9]. In the field of nephrology, CCI has been used to predict mortality of patients with acute kidney injury (AKI), diabetic kidney disease [10, 11]. As for patients with ESKD, several studies [12, 13] have validated that the CCI was an effective tool for comorbidity assessment and it could be used for survival prediction. To the best of our knowledge, there are few studies investigating the association between CCI and 30-day readmission in HD and PD patients. Therefore, we aimed to explore the association between CCI and the risk of 30-day readmission in patients receiving maintenance HD and PD based on a national administrative database in China.

## Methods

### Data sources and population setting

Patients on HD and PD therapy were extracted from the database of Hospital Quality Monitoring System (HQMS), which is a mandatory patient-level national database for hospital accreditation, under the authority of the Bureau of Medical Administration and Medical Service Supervision, National Health Commission of the People’s Republic of China. Details of the HQMS were described in detail elsewhere [14]. In brief, standardized electronic inpatient discharge summaries, which were completed within 48 h after hospital discharge, were uploaded on a daily basis to HQMS by most Class 3 hospitals (similar to tertiary hospitals) in China. The database collects 346 patient-level variables, including demographic characteristics, diagnoses, procedures, pathology diagnoses, and expenses. The discharge diagnoses were coded by professional medical coders at each hospital using the International Classification of Diseases-10 (ICD-10) coding system, and the procedures were coded using the International Classification of Diseases-9, Clinical Modification Volume 3 (ICD-9-CM-3).

The system was relatively well-established in 2013, and thus the data used in this study were from 1 January 2013 to 31 December 2015. The inclusion criteria for our study population included (1) aged ≥18 years old and ≤ 100 years old and (2) identified as receiving maintenance HD and PD by ICD-10 combined with ICD-9-CM-3. The ICD codes used for identifying dialysis patients are provided in Additional file 1: Table S1. The exclusion criteria included (1) patients without identification number and therefore readmissions could not be identified; (2) patients diagnosed as AKI, or chronic kidney disease (CKD) stage G1–4; (3) receipt of kidney transplantation during the index hospitalization; (4) patients died during the index hospitalization; and (5) patients’ records identified as having errors, e.g., the discharge date was earlier than admission date. Here we excluded those patients died during the index hospitalization because death is a competing risk for rehospitalization. There are specific diagnosis or procedure codes for PD, HD, AKI, CKD stages G1–4 and kidney transplantation respectively, and patients with all these diseases can be identified using corresponding ICD codes from the HQMS database. The ICD codes used for identifying patients with AKI, CKD stages G1–4 and kidney transplantation are provided in Additional file 1: Table S2.

The study outcome was the unplanned readmission following the index hospital discharge within 30 days. The planned readmission was identified by the specific question of “Is there any plan to readmit the patients within 30 days?” in the HQMS database, and those with positive answers were excluded. Furthermore, for those with a time interval of less than 2 days between two admissions were also excluded, because it might be a way to avoid a long length of stay, which is an indicator for healthcare quality evaluation. The reasons for readmission were considered in our analysis. We identified those readmission reasons using the medical procedure codes recorded during the rehospitalization first. The procedure codes were given the highest priority because the most important treatments for hospitalized patients can be identified through procedure codes. For those patients without recorded medical procedures during the rehospitalizations, we then used the first diagnosis as the causes of readmission. The recorded procedure and diagnosis codes of all included patients were classified into three categories: building dialysis access, dialysis comorbidity and other reasons, among which the first two are main causes of readmission with frequency > 10% respectively, and the third one consists of all those readmission reasons with frequency lower than 10%. We have listed the ICD codes of the readmission reasons in Additional file 1: Table S3.

**Fig. 1:**
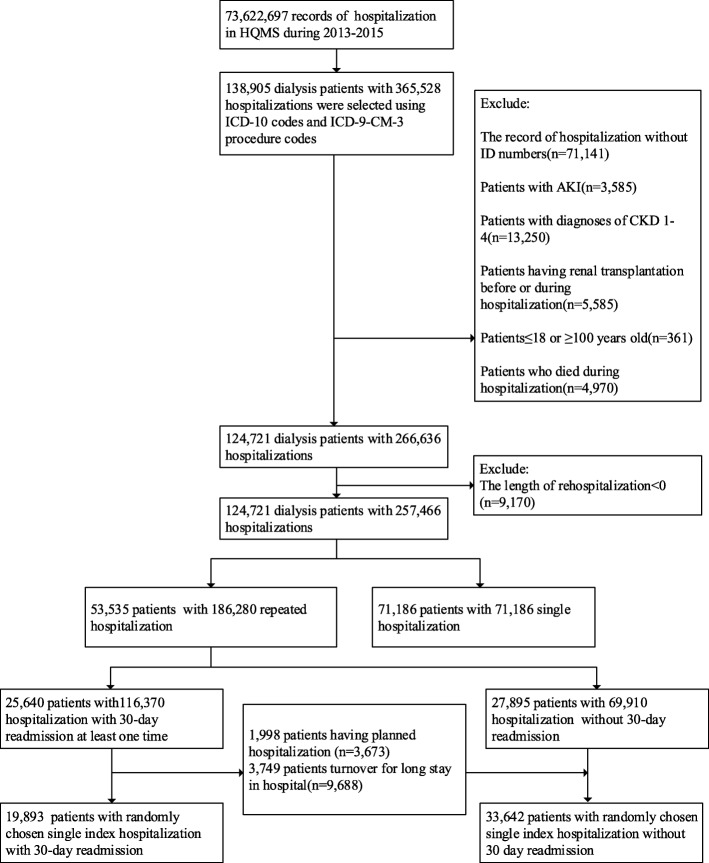
The procedure of study population selection

For patients with multiple hospitalizations, we randomly selected one hospitalization as the index hospitalization. Generally, compared with the first hospitalization, patients would have worse conditions in the later hospitalizations. To randomize the severity of patients’ illness, randomly selecting one hospitalization is a common approach to ensure the uniform distribution of the severity of patients’ conditions [6, 7]. The flowchart of the process for the patients’ inclusion is shown in Fig. 1.

### Comorbidities

Comorbidities were identified by ICD-10 coding (Additional file 1: Table S4). The first edition of CCI contains 19 comorbidities in which these comorbid diseases are weighted according to the severities of the corresponding chronic conditions. The second edition of CCI is a combined age-comorbidity score that adds the factor of age such that scores based on the second edition CCI are higher for older people [15] (The CCI with combined age-comorbidity is shown in Additional file 1: Table S5). In our study, the age-adjusted CCI was used because of its better predictive ability. More comorbidities and older age could result in a higher CCI score, which implies a higher disease burden. The CCI scores were classified into four groups based on the distribution in our study population: 2 (scored by ESKD per se), 3–4, 5–6, and > 6.

### Statistical analyses

The characteristics of patients included in the study were described. Data were reported as counts and percentages for categorical variables, and the mean (standard deviation, SD) or median (interquartile range, IQR) for continuous variables. Chi-square tests or Wilcoxon rank sum tests were used for comparison.

We calculated the 30-day readmission rate in different subgroups, grouped by the modality of dialysis and gender among dialysis patients with CCI score 2, 3–4, 5–6, and > 6. Patients were classified into different groups according to the causes of rehospitalization and the distribution of readmission reasons among patients in different CCI score categories were presented. A cause-specific Cox proportional hazards model was used to assess the risk of unplanned 30-day readmission. In addition to CCI, potential confounders or variables predictive of readmission were included in the multivariable models, including: sex (male or female), the length of hospital stay (continuous), mechanical ventilation (yes or no), resuscitation (yes or no), admission through emergency department (yes or no), modality of dialysis (hemodialysis or peritoneal dialysis), and admission to intensive care unit (ICU, yes or no). All the confounders in our analysis are clinically important variables. We considered that patients with longer length of hospital stay, mechanical ventilation, resuscitation, admission through emergency department, admission to ICU during their index hospitalizations are more likely to have severe condition and all these factors might have association with the risk of readmission [6, 16–18]. Since age was included in CCI, it was excluded from the proportional hazards model to avoid collinearity. Hazard ratios (HR) and 95% confidence intervals (CI) were reported. The proportional hazard assumptions were ascertained before fitting the Cox proportional hazard model.

All of the *P* values are 2-tailed. Python version 3.6 (Python Software Foundation, Delaware, United States) and R version 3.5.0 (R Foundation for Statistical Computing, Vienna, Austria) were used for analyses.

## Results

### General characteristics

Overall, 124,721 patients were included in the analysis. The characteristics of the dataset were described in Table 1. The mean age was 54.6 years old, and 41.5% were women. Regarding the modality of dialysis, 73.7% of patients were on hemodialysis.

**Table 1:** Characteristics of populations with and without 30-day readmission

Characteristic	Total *n* = 124,721	With 30-d Readmission *n* = 19,893	Without 30-d Readmission *n* = 104,828	*P*-value
Age (mean, SD)^b^	54.6 (15.6)	54.7 (15.4)	54.5 (15.7)	0.004
Women n(%)^a^	51,813 (41.5)	8166 (41.1)	43,647 (41.6)	0.125
Length of hospital stay (median, IQR)^b^	11 (11)	13 (14)	11 (12)	< 0.001
Mechanical ventilation n(%)^a^	584 (0.5)	41 (0.2)	543 (0.5)	< 0.001
ICU n(%)^a^	4043 (3.2)	496 (2.5)	3547 (3.4)	< 0.001
Rescue n(%)^a^	8088 (6.5)	1099 (5.5)	6989 (6.7)	< 0.001
Emergency n(%)^a^	17,904 (14.4)	2917 (14.7)	14,987 (14.3)	0.180
MI n(%)^a^	1123 (0.9)	130 (0.7)	993 (1.0)	< 0.001
Heart failure n(%)^a^	23,645 (19.0)	3899 (19.6)	19,746 (18.8)	0.012
Peripheral Vascular Disease n(%)^a^	1921 (1.5)	262 (1.3)	1659 (1.6)	0.006
Cerebrovascular disease n(%)^a^	10,376 (8.3)	1582 (7.6)	8794 (8.4)	0.042
Dementia n(%)^a^	149 (0.1)	27 (0.1)	122 (0.1)	0.540
Chronic Pulmonary Disease n(%)^a^	3355 (2.7)	489 (2.5)	2866 (2.7)	0.029
Connective Tissue Disease n(%)^a^	3547 (2.8)	617 (3.1)	2930 (2.8)	0.018
Peptic Ulcer Disease n(%)^a^	1178 (0.9)	195 (1.0)	983 (0.9)	0.593
Diabetes Mellitus uncomplicated n(%)^a^	20,963 (16.8)	3583 (18.0)	17,380 (16.6)	< 0.001
Mild Liver Disease n(%)^a^	7318 (5.9)	1069 (5.4)	6249 (6.0)	0.001
Hemiplegia n(%)^a^	153 (0.1)	23 (0.1)	130 (0.1)	0.842
Solid Tumor including leukemia, Lymphoma n(%)^a^	2726 (2.2)	360 (1.8)	2366 (2.3)	< 0.001
Diabetes Mellitus complicated n(%)^a^	20,724 (16.6)	3575 (18.0)	17,149 (16.4)	< 0.001
Moderate or Severe Liver Disease n(%)^a^	1733 (1.4)	250 (1.3)	1483 (1.4)	0.087
Characteristic	Total n = 124,721	With 30-d Readmission, n = 19,893	Without 30-d Readmission, n = 104,828	P-value
Solid Tumor with metastatic n(%)^a^	499 (0.4)	53 (0.3)	446 (0.4)	0.001
AIDS n(%)^a^	28 (< 0.1)	5 (< 0.1)	23 (< 0.1)	0.986
CCI (mean, SD)^b^	4.3 (2.0)	4.3 (2.0)	4.2 (2.0)	< 0.001
Modality of dialysis^a^				
Hemodialysis n(%)	91,901 (73.7)	12,760 (64.1)	79,141 (75.5)	< 0.001
Peritoneal dialysis n(%)	25,333 (20.3)	5493 (27.6)	19,840 (18.9)	
Unknown dialysis n(%)	7487 (6.0)	1640 (8.2)	5847 (5.6)	
Charlson Comorbidity Index^a^				
CCI = 2	30,413 (24.4)	4798 (24.1)	25,615 (24.4)	< 0.001
3 < =CCI < =4	42,051 (33.7)	6526 (32.8)	35,525 (33.9)	
5 < =CCI < =6	34,895 (28.0)	5674 (28.5)	29,221 (27.9)	
CCI > 6	17,371 (13.9)	2904 (14.6)	14,467 (13.8)	

*Abbreviations*: *AIDS* Acquired Immune Deficiency Syndrome, *CCI* Charlson Comorbidity Index, *ICU* intensive care unit, *MI* myocardial infarction, *SD* standard deviation, *IQR* interquartile range

^a^significance was assessed by Chi-squared test

^b^significance was assessed by Wilcoxon rank sum test

There were 19,893 patients identified as having unplanned 30-day readmission (16.0%). The median intervals between the date of discharge and the readmission was 20 days (IQR 14–21 days) among patients with 30-day readmission. Among the comorbidities included in CCI, heart failure, diabetes mellitus, and cerebrovascular disease were the three most frequent diseases in the study population. Compared with patients without readmission, a larger proportion of patients readmitted within 30 day were with higher CCI score, and the contrast was 28.5% vs. 27.9% (readmitted vs. non-readmitted, 5 < =CCI < =6) and 14.6% vs. 13.8% (readmitted vs. non-readmitted, CCI > 6), respectively. Furthermore, the percentage of patients receiving peritoneal dialysis was relatively higher among patients with readmission compared with those without (27.6% vs. 18.9%).

With the increase of CCI score, the 30-day readmission rates of patients grouped by the modality of dialysis and gender were shown in Table 2. For patients receiving hemodialysis, the readmission rate increased with elevated CCI scores. However, the trend was not observed for patients receiving peritoneal dialysis, who had relatively higher readmission rates compared with patients receiving hemodialysis.

**Table 2:** The 30-day readmission rate stratified by the Dialysis modality and gender in different CCI score groups

Subgroup	CCI = 2	3 < =CCI < =4	5 < =CCI < =6	CCI > 6	Total
Dialysis modality					
Hemodialysis	12.7%	13.3%	14.7%	15.3%	13.9%
Peritoneal	22.2%	21.2%	22.1%	20.9%	21.7%
Gender					
Male	15.8%	15.5%	16.5%	17.3%	16.1%
Female	15.7%	15.6%	16.0%	15.9%	15.8%
Total	15.8%	15.5%	16.3%	16.7%	16.0%

*Abbreviations*: *CCI* Charlson Comorbidity Index

The distribution of readmission reasons among different CCI score groups was shown in Table 3. Most patients (68.8%) were readmitted for other reasons instead of causes related to dialysis. For those patients readmitted because of dialysis related reasons, dialysis comorbidity is a more frequent cause than building dialysis access (20.1% vs. 11.1%). The distribution of readmission reasons among patients in different CCI score groups has no significant difference (*P* > 0.05).

**Table 3:** The distribution of readmission reasons among patients in different CCI score groups

Reason of rehospitalization	CCI = 2 n (%)	3 = <CCI < =4 n (%)	5 = <CCI < =6 n (%)	CCI > 6 n (%)	Total n (%)	*P*-value^a^
Dialysis access	531 (11.1)	698 (10.7)	659 (11.6)	313 (10.8)	2201 (11.1)	0.3
Dialysis complications	920 (19.2)	1355 (20.8)	1145 (20.2)	580 (20.0)	4000 (20.1)	
Others	3338 (69.7)	4473 (68.5)	3870 (68.2)	2011 (69.3)	13692 (68.8)	

*Abbreviations*: *CCI* Charlson Comorbidity Index

^a^significance was assessed by Chi-squared test

### CCI as the predictor of rehospitalization within 30 days

Categories of CCI were independently associated with the risk of unplanned 30-day readmission (Table 4). In the fully adjusted model, compared with patients with CCI of 2, the HR for those with CCI 3–4, 5–6 and > 6 was 1.01 (95% CI 0.98–1.05), 1.09 (95% CI 1.05–1.14), and 1.14 (95% CI 1.09–1.20), respectively.

**Table 4:** Association between CCI and 30-day readmission adjusted for gender, dialysis modality and other variables, respectively

CCI Subgroup	Gender-adjusted HR	Gender and dialysis modality-adjusted HR	Multivariable-adjusted HR^a^
CCI = 2	1	1	1
3 < =CCI < =4	0.99 (0.95,1.01)	1.01 (0.97,1.05)	1.01 (0.98,1.05)
5 < =CCI < =6	1.04 (1.00,1.08)	1.09 (1.05,1.14)	1.09 (1.05,1.14)
CCI > 6	1.07 (1.02,1.12)	1.14 (1.09,1.19)	1.14 (1.09,1.20)

*Abbreviations*: *CCI* Charlson Comorbidity Index, *HR* hazard ratio

^a^Adjusted for gender, the length of hospital stay, the receipt of mechanical ventilation, rescue, admission through emergency, the modality of dialysis and admission to intensive care unit (ICU)

## Discussion

To the best of our knowledge, this study is the first describing the association between CCI and unplanned 30-day readmission among patients receiving maintenance dialysis based on a large nationwide database. The unplanned 30-day readmission rate in our study was 16.0%. Furthermore, CCI was independently associated with the risk of 30-day readmission and could therefore be used for risk stratification for hospitalized dialysis patients. Besides, we randomly selected one hospitalization as the index hospitalization, and the CCI score of patients in the index hospitalization could be useful to stratify a patient’s risk of readmission according to our result. Patients on dialysis may have uniform distribution of illness severity in their randomly selected index hospitalizations and CCI score can play as an important predictor for readmission.

The 30-day readmission rate for patients with kidney disease varied across different countries. The proportion of 30-day readmission in ESKD patients was 35.4% reported by the US Renal Data System (USRDS), while it was 17.0% of patients receiving hemodialysis from 157 acute care hospitals in Canada, which is similar to our results [6, 19]. Possible reasons for the variation might include differences in the pattern of dialysis service and patients’ characteristics. Furthermore, the 30-day readmission rate in the general patient populations might contribute substantially to the variation. For example, the readmission rate within 30 days was 15.3% among Medicare beneficiaries without CKD in the US, and it was 8.5% among overall hospitalized patients in Canada [6, 19]. In China, the readmission rate among ESKD patients was also two times that of all in-patients (10% versus 5.18%) in 2015[20], and was much higher compared with patients with diabetes (2.4–4.2% during the year 2008–2013) [21] or patients with acute exacerbation for chronic obstructive pulmonary disease (COPD) (6.8%) [22]. Therefore, although varied, high readmission rates among the ESKD population were common and imposed increased burdens on health systems.

Furthermore, our results revealed that the readmission rate was higher among patients on peritoneal dialysis compared to those on hemodialysis, which is consistent with previous reports. Perl et al.[23] demonstrated that patients on peritoneal dialysis had a 19% higher readmission risk than patients receiving hemodialysis therapy. Lafrance et al. [24] found that the modality of peritoneal dialysis was associated with an increased risk of infection-related hospitalizations compared with the use of hemodialysis [24]. For patients on peritoneal dialysis, peritonitis and bacteremia were common complications, which may have increased the risk of infection-related disease, and fewer physician visits in the dialysis unit than in hemodialysis patients probably resulted in the higher readmission rate.

Previous studies investigated the association between CCI and mortality among various types of kidney disease, and all of these studies suggested that the CCI could be used as a predictor of adverse outcome for patients with kidney disease [10, 11, 13]. Some studies developed modified CCIs [12, 25, 26] for mortality analysis of dialysis patients without counting kidney disease, but the performance of a modified CCI is almost identical to the original CCI in terms of c-statistics. In our study, although the overall CCI score is systematically deviated towards a higher score as kidney disease is scored for patients receiving dialysis. The deviated CCI score of patients on dialysis would not affect the relationship between CCI and unplanned readmission because we transformed a numerical CCI score to a categorical one by classifying patients into four different groups. Furthermore, there were other comorbidity indexes developed for patients receiving maintenance dialysis. For example, Wright-Khan [27] proposed a comorbidity index based on data of ESKD patient. Davies [28] developed a comorbidity index to predict the mortality of patients on ambulatory peritoneal dialysis. Several studies compared the above two indexes with CCI, and found that the CCI had the best performance regarding prediction of mortality [29, 30]. However, there are few studies investigating the association between comorbidity-indexes and the risk of 30-day readmission among patients with ESKD. CCI was initially designed to predict 1-year mortality for all hospitalized patients, but it has never been used to perform 30-day readmission prediction in dialysis patients. Previous studies have explored the association between CCI and readmission in the hip fracture population [31] and in patients after orthopedic surgery [32]. However, the predictive abilities of CCI for readmission in different populations were inconsistent. Our study indicated that a higher CCI score may indicate a higher risk of 30-day readmission rate, and it could therefore be used for risk stratification in clinical practice.

This study has the advantage of utilizing a large nationwide database with strict quality-control processes. However, the study has some limitations worth mentioning. First, although a national database was used to explore the association between CCI and the risk of 30-day readmission, it included only class 3 hospitals and lacked hospitalization records of dialysis patients from the primary and secondary hospitals. Selection bias might have occurred because the patients in class 3 hospitals tend to have severe situations which probably overestimated the readmission rate. However, the class 3 hospitals included in the HQMS could provide healthcare services to nationwide patients due to the lack of a standard referral system. Hospitalizations for all causes were considered in our study, as not all dialysis patients were admitted through nephrology in China. Second, we selected the patients on maintenance dialysis based on the diagnosis code of ICD-10 and the procedure code of ICD-9-CM-3, which may have ignored those patients with conditions of dialysis but having no records of the dialysis diagnosis or procedure in the database. However, the use of ICD codes to extract patients from an administrative or claims dataset is a common method for observational study. In addition, a previous study of HQMS [14] has shown that ICD codes in the database had relatively low sensitivity and high specificity. According to the results, the non-dialysis patients were less likely to be misclassified as dialysis patients, which ensured the homogeneity of our population. Finally, as an administrative database, the database of HQMS lacked the variables of vital signs, results of laboratory tests and clinical medications, which implied that there may be residual confounding. Considering that these variables were not recorded in inpatient discharge summaries, and that we aimed to predict the 30-day readmission of dialysis patients from routinely available data, the use of HQMS was the best choice at present. The HQMS database has been used in previous studies [14, 33] to analyze the distribution and trend of CKD in China and the utilization of this dataset for rehospitalization research is also feasible.

## Conclusions

Our study indicated that CCI was a predictor for the risk of 30-day readmission among patients with ESKD, and could be used for clinical risk prediction and patient management. For hospitalized dialysis patients with a CCI of 5 or higher, relatively intense follow-up is needed, especially during the first month after discharge. Whether the effective risk prediction and corresponding intervention could lead to the reduction of 30-day readmission, as well as medical expenditures, warrants further interventional research.

## Additional file

Additional file 1: **Table S1.** The ICD-10 diagnosis codes and ICD-9-CM-3 procedure codes for identifying dialysis patients. **Table S2.** The ICD-10 diagnosis codes and ICD-9-CM-3 procedure codes for excluding patients with AKI, kidney transplantation and CKD stages G1–4. **Table S3.** The ICD-10 diagnosis codes and ICD-9-CM-3 procedure codes for identifying causes of hospitalization. **Table S4.** The ICD-10 diagnosis codes for identifying the diseases in CCI. **Table S5.** Comorbidity component of CCI and weighted score. (DOCX 19 kb)

## Data Availability

The data that support the findings of this study are available from the Bureau of Medical Administration and Medical Service Supervision, National Health Commission of China but restrictions apply to the availability of these data, which were used under license for the current study, and so are not publicly available. Data are however available from the authors upon reasonable request and with permission of the Bureau of Medical Administration and Medical Service Supervision, National Health Commission of China.

## Abbreviations

AKI: Acute Kidney Injury
CCI: Charlson Comorbidity Index
CI: Confidence interval
CKD: Chronic kidney disease
ESKD: End-stage kidney disease
HQMS: Hospital Quality Monitoring System
HR: Hazard ratios
ICD: The International Classification of Diseases
ICD-10: The International Classification of Diseases-10 coding system
ICD-9-CM-3: The International Classification of Diseases-9, Clinical Modification Volume 3
ICU: Intensive care unit
IQR: Interquartile range
SD: Standard deviation
USRDS: US Renal Data System

## References

[1] Liyanage T, Ninomiya T, Jha V, Neal B, Patrice HM, Okpechi I (2015). Worldwide access to treatment for end-stage kidney disease: a systematic review. Lancet.

[2] Saran R, Robinson B, Abbott KC, Agodoa LYC, Bhave N, Bragg-Gresham J (2018). US renal data dystem 2017 annual data report: Epidemiology of kidney disease in the United States. Am J Kidney Dis.

[3] Fishbane S, Wish JB (2016). Quality measurement in Wonderland: The curious case of a dialysis readmissions measure. Clin J Am Soc Nephrol.

[4] US Government Printing Office. Federal Register. In: U.S. Government Publishing Office. https://www.gpo.gov/fdsys/pkg/FR-2014-11-06/pdf/2014–26182.pdf; Accessed 12 Jan 2015.

[5] Hakim RM, Collins AJ (2014). Reducing avoidable rehospitalization in ESRD: a shared accountability. J Am Soc Nephrol.

[6] Harel Z, Wald R, McArthur E, Chertow GM, Harel S, Gruneir A (2015). Rehospitalizations and emergency department visits after hospital discharge in patients receiving maintenance hemodialysis. J Am Soc Nephrol.

[7] Flythe JE, Katsanos SL, Hu Y, Kshirsagar AV, Falk RJ, Moore CR (2016). Predictors of 30-day hospital readmission among maintenance hemodialysis patients: a hospital’s perspective. J Am Soc Nephrol.

[8] Charlson ME, Pompei P, Ales KL, MacKenzie CR (1987). A new method of classifying prognostic comorbidity in longitudinal studies: development and validation. J Chronic Dis.

[9] de Groot V, Beckerman H, Lankhorst GJ, Bouter LM (2003). How to measure comorbidity: a critical review of available methods. J Clin Epidemiol.

[10] Talib S, Sharif F, Manzoor S, Yaqub S, Kashif W (2017). Charlson comorbidity index for prediction of outcome of acute kidney injury in Critically Ill Patients. Iran J Kidney Dis.

[11] Huang YQ, Gou R, Diao YS, Yin QH, Fan WX, Liang YP (2014). Charlson comorbidity index helps predict the risk of mortality for patients with type 2 diabetic nephropathy. J Zhejiang Univ Sci B.

[12] Hemmelgarn BR, Manns BJ, Quan H, Ghali WA (2003). Adapting the Charlson Comorbidity index for use in patients with ESRD. Am J Kidney Dis.

[13] Rattanasompattikul M, Feroze U, Molnar MZ, Dukkipati R, Kovesdy CP, Nissenson AR (2012). Charlson comorbidity score is a strong predictor of mortality in hemodialysis patients. Int Urol Nephrol.

[14] Huang YM, Xu D, Long J, Shi Y, Zhang L, Wang H (2018). The spectrum of chronic kidney disease in China: a national study based on hospitalized patients from 2010 to 2015. Nephrology.

[15] Charlson M, Szatrowski TP, Peterson J, Gold J (1994). Validation of a combined comorbidity index. J Clin Epidemiol.

[16] Hua M, Gong MN, Brady J, Wunsch H (2015). Early and late unplanned rehospitalizations for survivors of critical illness*. Crit Care Med.

[17] Low LL, Liu N, Wang S, Thumboo J, Ong ME, Lee KH (2016). Predicting 30-Day Readmissions in an Asian Population: Building a Predictive Model by Incorporating Markers of Hospitalization Severity. PloS one.

[18] Reynolds K, Butler MG, Kimes TM, Rosales AG, Chan W, Nichols GA (2015). Relation of Acute Heart Failure Hospital Length of Stay to Subsequent Readmission and All-Cause Mortality. Am J Cardiol.

[19] Saran R, Robinson B, Abbott KC, Agodoa LYC, Bragg-Gresham J, Balkrishnan R (2018). US Renal Data System 2017 Annual Data Report Epidemiology of Kidney Disease in the United States. Am J Kidney Dis.

[20] National Health and Family Planning Commission (2016). National Medical Service and Quality Safety Report.

[21] Liu X, Guo Y, Li D, Cui Z, Liu Y, Li C (2017). The prevalence and long-term variation of hospital readmission for patients with diabetes in Tianjin, China: A cross-sectional study. Medicine.

[22] Lin J, Xu Y, Wu X, Chen M, Lin L, Gong L (2014). Risk factors associated with chronic obstructive pulmonary disease early readmission. Curr Med Res Opin.

[23] Perl J, McArthur E, Bell C, Garg AX, Bargman JM, Chan CT (2017). Dialysis Modality and Readmission Following Hospital Discharge: A Population-Based Cohort Study. Am J Kidney Dis.

[24] Lafrance JP, Rahme E, Iqbal S, Elftouh N, Vallee M, Laurin LP (2012). Association of dialysis modality with risk for infection-related hospitalization: a propensity score-matched cohort analysis. Clin J Am Soc Nephrol.

[25] Liu JN, Huang Z, Gilbertson DT, Foley RN, Collins AJ (2010). An improved comorbidity index for outcome analyses among dialysis patients. Kidney Int.

[26] Chen JY, Tsai SH, Chuang PH, Chang CH, Chuang CL, Chen HL (2014). A comorbidity index for mortality prediction in Chinese patients with ESRD receiving hemodialysis. Clin J Am Soc Nephrol.

[27] Khan IH, Catto GR, Edward N, Fleming LW, Henderson IS, MacLeod AM (1993). Influence of coexisting disease on survival on renal-replacement therapy. Lancet.

[28] Davies SJ, Russell L, Bryan J, Phillips L, Russell GI (1995). Comorbidity, Urea Kinetics, And Appetite In Continuous Ambulatory Peritoneal-Dialysis Patients - Their Interrelationship And Prediction Of Survival. Am J Kidney Dis.

[29] van Manen JG, Korevaar JC, Dekker FW, Boeschoten EW, Bossuyt PMM, Krediet RT (2002). How to adjust for comorbidity in survival studies in ESRD patients: A comparison of different indices. Am J Kidney Dis.

[30] Fried L, Bernardini J, Piraino B (2001). Charlson comorbidity index as a predictor of outcomes in incident peritoneal dialysis patients. Am J Kidney Dis.

[31] Toson B, Harvey LA, Close JC (2015). The ICD-10 Charlson Comorbidity Index predicted mortality but not resource utilization following hip fracture. J Clin Epidemiol.

[32] Voskuijl T, Hageman M, Ring D (2014). Higher Charlson Comorbidity Index Scores are associated with readmission after orthopaedic surgery. Clin Orthop Relat Res.

[33] Zhang L, Wang H, Long J, Shi Y, Bai K, Jiang W (2017). China Kidney Disease Network (CK-NET) 2014 Annual Data Report. Am J Kidney Dis.

