# Supplementary material for: Association between the Charlson Comorbidity Index and the risk of 30-day unplanned readmission in patients receiving maintenance dialysis
Source: BMC Nephrol. 2019 Oct 7;20:363. doi: 10.1186/s12882-019-1538-0 (PMC6781396; doi:10.1186/s12882-019-1538-0)
Supplement: Supplementary file 1 — Table S1. The ICD-10 diagnosis codes and ICD-9-CM-3 procedure codes for identifying dialysis patients. Table S2. The ICD-10 diagnosis codes and ICD-9-CM-3 procedure codes for excluding patients with AKI, kidney transplantation and CKD stages G1–4. Table S3. The ICD-10 diagnosis codes and ICD-9-CM-3 procedure codes for identifying causes of hospitalization. Table S4. The ICD-10 diagnosis codes for identifying the diseases in CCI. Table S5. Comorbidity component of CCI and weighted score. (DOCX 19 kb) [file 12882_2019_1538_MOESM1_ESM.docx]

###### Appendix

**Table S1 The ICD-10 diagnosis codes and ICD-9-CM-3 procedure codes for identifying dialysis patients**

| **Dialysis** | **Diagnosis codes of ICD-10** | | **Procedure codes of ICD-9-CM-3(Beijing version)** |
| --- | --- | --- | --- |
|  | National version | Beijing Version |  |
| **Hemodialysis** | T80.801, T80.902, T82.400, T82.401, Z99.201 | T82.401, Z99.201 | 38.93003, 38.93004, 38.95001, 39.27001, 39.27002, 39.42001, 39.43001, 39.49004, 39.95003 |
| **Peritoneal dialysis** | T85.609, T85.610, T85.611, T85.710, T85.711, T85.801, T85.901, Z49.201 | T80.201, T85.602, Z49.201, Z99.202 | 54.93002, 54.98001, 97.86003 |
|  |  |  |  |
| **Unknown type of dialysis** | Z49.101, Z49.200, Z99.200 | Z49.101, Y84.101 |  |

Note: There is a little difference in diagnosis codes of different ICD-10 version. Some regions use ICD-10 of Beijing version while the other regions use ICD-10 of national version in China.

**Table S2 The ICD-10 diagnosis codes and ICD-9-CM-3 procedure codes for excluding patients with AKI, kidney transplantation and CKD stages G1-4**

| **Disease** | **Diagnosis codes of ICD-10** | | **Procedure codes of ICD-9-CM-3(Beijing version)** |
| --- | --- | --- | --- |
|  | National version | Beijing Version |  |
| **Acute kidney injury** | N17, N01, T79.5, D59.3, K76.7, O90.4, N96.x00, A23.100, O00.101, O00.105, O00.108, O00.111, O00.114, O02.001, O02.100, O03, O04, O05, O06.900, O07, O08 (exclude O08.006, O08.103, O08.104, O08.105, O08.106, O08.302, O08.806), O20.000, O26.200, O31.100, Z09.802, Z35.100, Z35.101, Z35.104, N10.x00, N10.x01, N99.000, N99.001 | N17, N01, T79.5, D59.3, K76.7, O90.4, N96xx01, , O00.102, O00.107, O00.110, O00.111, O02.101, O03, O04(exclude O04.905, O04.907), O05, O06(exclude O06.907, O06.908) O07, O08(exclude O08.103, O08.105, O08.301, O08.801, O08.803, O08.806, O08.807, O08.809) O20.001, O26.201, Z30.201, Z35.102, Z98.8308, Z98.8312, N00.908, N10xx03, N10xx04+H20.9* |  |
| **Kidney transplantation** | Z94.000, Z94.002, T86.100, T86.101, T86.102, T86.103, T86.104, T86.105, T86.106, T86.100, N99.804, T82.802 | Z94.002, Z09.002, T86.101, T86.102 | 50.59001, 50.59004, 55.01007, 55.53001, 55.61002, 55.69001, 55.86004, 55.89002, 55.92006 |
| **CKD stage G1-4** | N18.801,N18.802,N18.803,N18.804,N18.001 | N18.914,N18.915,N18.916,N18.917,N18.918 |  |

**Table S3 The ICD-10 diagnosis codes and ICD-9-CM-3 procedure codes for identifying causes of hospitalization**

| **cause of hospitalization** | **Diagnosis codes of ICD-10** | | **Procedure codes of ICD-9-CM-3(Beijing version)** |
| --- | --- | --- | --- |
|  | National version | Beijing Version |  |
| **Dialysis access** | I97.804 | I77.003, Z99.201 | 39.27001, 38.95001, 38.93004, 38.93003, 39.27002, 39.29025, 54.93002 |
| **Dialysis comorbidity** | T85.703, I97.806, I97.807, T82.400, I97.805, T82.401, T80.902, T81.702, T82.809, T82.811, T82.800, T82.700, T80.801, Z45.200, T82.808, T82.702, T82.810, T82.804, T82.500, T82.807, T82.902, T85.711, T85.710, T85.611, T85.609, T85.610, T85.901, T85.700, T85.801 | T82.703, T82.401, T82.810, T82.802, T82.804, T82.901, T82.805, T82.809, E89.801, T82.808, T82.806, T85.602 | 39.42001, 39.49004, 88.51001, 39.53002, 39.53001, 39.43001, 39.53003, 39.50019, 38.03003, 38.03002, 38.63002, 39.53007, 38.33001, 39.50018, 39.49006, 39.49001, 39.50021, 38.03001, 38.30001, 38.83004, 39.50017, 38.43001, 38.83003, 38.63001, 38.65001, 97.86003 |
|  |  |  |  |

**Table S4 The ICD-10 diagnosis codes for identifying the diseases in CCI**

| Disease | ICD-10 |
| --- | --- |
| Myocardial Infarction | I21, I22, I23, I24 |
| Congestive Heart Failure | I50 |
| Peripheral Vascular Disease | I73, I77, K55 |
| Cerebrovascular Disease | G45, G46, I60, I61, I62, I63, I64, I65, I66, I67, I68, I69 |
| Dementia | F00, F01, F02, F03, G30 |
| Chronic Pulmonary Disease | J40, J41, J43, J44, J45, J46, J47, J60, J61, J62, J63, J64, J65, J66, J67, J68, J69, J70 |
| Connective Tissue Disease | M05, M06, M08, M30, M31, M32, M33, M34, M35, M36 |
| Peptic Ulcer Disease | K25, K26, K27, K28 |
| Diabetes Mellitus uncomplicated | E10.9, E11.9, E12.9, E13.9, E14.9 |
| Mild Liver Disease | B18, K70, K73, K75, K76 |
| Hemiplegia | G81, G82, G83 |
| Moderate to Severe Chronic Kidney Disease | all patients |
| Solid Tumor without metastatic, including leukemia and lymphoma | C0, C1, C2, C3, C4, C5, C6, C71, C9, C70, C72, C73, C74, C75, C76, C81, C82, C83, C84, C85, C88 |
| Diabetes Mellitus complicated | E10.0, E10.1, E10.2, E10.3, E10.4, E10.5, E10.6, E10.7, E10.8, E11.0, E11.1, E11.2, E11.3, E11.4, E11.5, E11.6, E11.7, E11.8, E12.0, E12.1, E12.2, E12.3, E12.4, E12.5, E12.6, E12.7, E12.8, E13.0, E13.1, E13.2, E13.3, E13.4, E13.5, E13.6, E13.7, E13.8 E14.0, E14.1, E14.2, E14.3, E14.4, E14.5, E14.6, E14.7, E14.8 |
| Moderate or Severe Liver Disease | K71, K72, K74, I85 |
| Solid Tumor with metastatic | C77, C78, C79, C80 |
| AIDS | B20, B21, B22, B23, B24 |

Note: The diagnosis codes for identifying the disease in CCI mainly referred to the codes of ICD-10 developed by Sundararajan ^[1]^ which was widely used in the literature. As the ICD-10 system is country specific, the codes of ICD-10 used to ascertain comorbidities of CCI in Korea ^[2]^ was also referred to find out the suitable diagnosis codes corresponding to dialysis patients in our database.

**Table S5 Comorbidity component of CCI and weighted score**

| Comorbidity | Score |
| --- | --- |
| Myocardial Infarction | 1 |
| Congestive Heart Failure | 1 |
| Peripheral Vascular Disease | 1 |
| Cerebrovascular Disease | 1 |
| Dementia | 1 |
| Chronic Pulmonary Disease | 1 |
| Connective Tissue Disease | 1 |
| Peptic Ulcer Disease | 1 |
| Mild Liver Disease | 1 |
| Diabetes Mellitus, uncomplicated | 1 |
| Moderate to Severe Chronic Kidney Disease | 2 |
| Hemiplegia | 2 |
| Diabetes Mellitus, complicated | 2 |
| Cancer (Leukemia, Malignant Lymphoma, Solid Tumor) | 2 |
| Moderate or Severe Liver Disease | 3 |
| Metastatic Solid Tumor | 6 |
| AIDS | 6 |

For each decade, CCI should add 1 point over age of 50, up to 3 points

Reference

1 Sundararajan V., Henderson T., Perry C., et al. New ICD-10 version of the Charlson comorbidity index predicted in-hospital mortality [J]. J Clin Epidemiol, 2004, 57(12): 1288-94.

2 Chae J. W., Song C. S., Kim H., et al. Prediction of Mortality in Patients Undergoing Maintenance Hemodialysis by Charlson Comorbidity Index Using ICD-10 Database [J]. Nephron Clin Pract, 2011, 117(4): C379-C84.
